# When should a laparoscopic cholecystectomy be performed after ERCP for common bile duct stones combined with gallbladder stones? A meta-analysis of randomized controlled trials

**DOI:** 10.3389/fsurg.2026.1889144

**Published:** 2026-10-07

**Authors:** Tingfei Shi, Chunqing Fu, Yupeng Li, Wu Chen, Haibo Sun, Yiyu Jin, Xianke Lin, Wenting Cao, Bo Ran

**Affiliations:** 1 The Second Affiliated Hospital of Hainan Medical University, Haikou, Hainan, China; 2 Hainan Medical University, Haikou, Hainan, China; 3 School of Public Health, Key Laboratory of Tropical Translational Medicine of Ministry of Education, Hainan Medical University, Haikou, Hainan, China

**Keywords:** common bile duct stones, endoscopic retrograde cholangiopancreatography, gallbladder stones, laparoscopic cholecystectomy, meta-analysis

## Abstract

**Background:**

Gallstones combined with common bile duct stones are a common disease in biliary surgery. At present, for gallbladder stones with common bile duct stones, the globally recognized standard treatment is a step-by-step treatment of “endoscopy combined with surgery”. First, more urgent and potentially life-threatening bile duct stones are treated, and then gallbladder stones are treated. However, after the common bile duct stones are successfully removed by ERCP, how long should laparoscopic cholecystectomy be performed at intervals? There is a dispute between “early” (defined as within 72 h after ERCP) and “delayed” (defined as 72 h after ERCP). The conclusion is not yet uniform.

**Objective:**

To systematically analyze the efficacy and safety of early LC and delayed LC in patients with gallstones and common bile duct stones after ERCP, to provide current high-level evidence-based medical evidence for clinical decision-making, to help clinicians optimize the diagnosis and treatment process, to reduce the occurrence of adverse complications caused by treatment.

**Method:**

PubMed, Embase, Cochrane Library, Web of Science, Scopus, and other databases were searched to collect randomized controlled trials (RCTs) comparing early LC and delayed LC after ERCP from inception to December 2025. Two reviewers independently screened literature, extracted data, and assessed the risk of bias of included studies using the Cochrane ROB evaluation tool. The RevMan 5.4 software was used to analyze the outcomes of each group. Unlike previous meta-analyses, this study only included randomized controlled trials and incorporated the latest ones. Moreover, the quality of each outcome indicator was evaluated using the GRADE method.

**Result:**

This meta-analysis included 7 randomized controlled trials involving 593 patients (296 in the early LC group and 297 in the delayed LC group). Compared with the delayed LC group, the early LC group was associated with a significantly lower rate of conversion to open surgery (*RR* = 0.35, 95*% CI*: 0.17–0.68, *P* = 0.002), shorter operation time (*MD* = −14.37 min, 95*% CI*: −28.22 to −0.51, *P* = 0.04), shorter postoperative hospital stay (*MD* = −0.81 days, 95*% CI*: −1.20 to −0.42, *P* < 0.0001), and lower incidence of incision infection *(RR* = 0.21, 95*% CI*: 0.07–0.61, *P* = 0.004). No significant difference was observed between the two groups in the incidence of bile leakage (*RD* = −0.04, 95*% CI*: −0.12–0.04, *P* = 0.30).

**Conclusion:**

In conclusion, the available RCT evidence suggests that early LC following successful ERCP may provide clinical advantages over delayed LC, including lower conversion and infection rates and shorter operative and postoperative hospital stay. However, because of outcome-level heterogeneity, the limited number of included trials, and few bile-leakage events, these findings should be interpreted with caution and further verified in larger well-designed RCTs.

**Systematic Review Registration:**

https://www.crd.york.ac.uk/PROSPERO/view/CRD420261395564, PROSPERO CRD420261395564.

## 1 Introduction

Gallstones combined with common bile duct stones are a common disease in biliary surgery, accounting for about 10%–15% of patients with gallstones (1). For such patients, the best treatment has been controversial. At present, the two-stage treatment strategy-first endoscopic retrograde cholangiopancreatography and sphincterotomy, and then laparoscopic cholecystectomy, has been widely used in various countries (2). However, there are still differences in clinical practice regarding the optimal interval between ERCP and LC. The traditional view advocates a delay of 6–8 weeks of surgery, waiting for “inflammation to subside and patients to fully recover” (3); some studies suggest that early surgery should be performed to reduce the risk of recurrent biliary events during the waiting period (4, 5).

In recent years, a number of randomized controlled trials have compared the clinical outcomes of different LC timings, but single studies have failed to reach a positive conclusion due to limited sample size (4, 6–11). Therefore, this study used a meta-analysis method to systematically evaluate the efficacy and safety of early LC and delayed LC after ERCP, aiming to provide evidence-based medical evidence for clinical treatment decision-making.

## 2 Materials and methods

This study was performed in accordance with the Cochrane Review and Preferred Reporting Items for Systematic Review and Meta-Analysis Protocols guidelines (12, 13). The study protocol was registered with the PROSPERO (registration number: CRD420261395564).

### 2.1 Search strategy

This study aims to systematically review the literature related to “laparoscopic cholecystectomy following ERCP in patients with gallbladder and common bile duct stones.” A computerized search was conducted in databases including PubMed, Embase, the Cochrane Library, Web of Science, and Scopus, with the search period spanning from the inception of each database to December 15, 2025. A combination of subject headings and free-text terms was used. English search terms included: Choledocholithiasis, Common Bile Duct Stones, Gallstones, Cholelithiasis, ERCP, Cholecystectomy, Laparoscopic Cholecystectomy, Early, Delayed, Timing, randomized controlled trial, etc. For example, the Scopus search query is: TITLE-ABS-KEY (Choledocholithiasis OR “Common Bile Duct Stones”) AND TITLE-ABS-KEY (Gallstones OR Cholelithiasis) AND TITLE-ABS-KEY (ERCP OR “Endoscopic Retrograde Cholangiopancreatography” OR “Endoscopic Retrograde Cholangiopancreatography”) AND TITLE-ABS-KEY (Cholecystectomy OR “Laparoscopic Cholecystectomy”) AND TITLE-ABS-KEY (Early OR Delayed OR Timing) AND TITLE-ABS-KEY (“Randomized Controlled Trial” OR RCT. Search strategies for other databases were similar to the examples provided. Additionally, one reference from a meta-analysis (11) was manually identified and included in this study.

### 2.2 Inclusion and exclusion criteria

#### 2.2.1 Study type

Published randomized controlled trials, whether blinded or not, must be in English.

#### 2.2.2 Object of study

(1) Diagnosis of gallbladder stones with concomitant common bile duct stones confirmed by imaging or ERCP; (2) Successful completion of ERCP/EST and removal of stones from the common bile duct; (3) No contraindications for laparoscopic cholecystectomy; (4) Exclusion of acute pancreatitis, acute cholangitis, or acute cholecystitis requiring emergency surgery.

#### 2.2.3 Intervening measure

The experimental group consisted of patients undergoing early LC, defined as LC performed within 72 h of ERCP; the control group consisted of patients undergoing delayed LC, defined as LC performed more than 72 h after ERCP. When available, the actual delayed-LC timing reported in each original trial was extracted and summarized in Table 1; when the original report did not provide a more precise interval, this was recorded as not reported (NR).

**Table 1:** General characteristics of the included studies.

Study	Country	Yr	Sample size (E/D)	Definition of early LC	Definition of delayed LC	Primary outcomes	Actual delayed-LC timing reported
Salman B	Turkey	2009	39/40	≤72 h	>72 h	① Conversion rate ② Operative time ③ Length of hospital stay	Reported as >72 h; exact interval NR
Reinders JSK	Netherlands	2010	47/47	≤72 h	>72 h	① Conversion rate ② Surgical site infection ③ Bile leak	6–8 weeks after ES/ERCP
El Nakeeb A	Egypt	2016	55/55	≤72 h	>72 h	①Conversion rate ② Bile leak	1 month after ERCP
Ali RF	Egypt	2021	20/20	≤72 h	>72 h	① Operative time ② Length of hospital stay ③ Surgical site infection	6 weeks after ERCP
Mathur A	India	2021	35/35	≤72 h	>72 h	① Conversion rate ② Operative time ③ Length of hospital stay	6 weeks after ERCP
Godara D	India	2021	75/75	≤72 h	>72 h	① Conversion rate ② Operative time ③ Length of hospital stay	Reported as >72 h; exact interval NR
Reddy MMM	India	2024	25/25	≤72 h	>72 h	① Operative time ② Length of hospital stay ③ Surgical site infection	4 weeks after ERCP

E, early laparoscopic cholecystectomy group; D, delayed laparoscopic cholecystectomy group; LC, laparoscopic cholecystectomy; h, hours; NR, not reported in detail in the original report.

#### 2.2.4 Outcome indicator

(1) Primary outcome: rate of conversion to open surgery; (2) Secondary outcomes: operative time, length of hospital stay, postoperative incisional infection rate, and postoperative bile leak rate.

#### 2.2.5 Exclusion criteria

(1) Studies that are not randomized controlled trials (RCTs); (2) Duplicate publications; (3) Studies with incomplete data or from which valid data cannot be extracted.

### 2.3 Literature screening and data extraction

Two researchers independently screened the literature, extracted data, and cross-checked their findings. Any discrepancies were resolved through discussion or consultation with a third party. The extracted information included: (1) basic information: first author, year of publication, and country; (2) study population: sample size; (3) intervention: specific time definitions for early LC and delayed LC, including the actual delayed-LC interval when reported; and (4) outcome data.

### 2.4 Risk assessment of bias

The Cochrane ROB tool, recommended by the Cochrane Handbook of Systematic Reviews, was used to assess the risk of bias in RCTs. This assessment included the following domains: random sequence generation, allocation concealment, blinding, completeness of outcome data, reporting bias, and other sources of bias. Each domain was rated as “low risk,” “high risk,” or “unclear.”

### 2.5 Statistical analysis

Meta-analyses were conducted using RevMan 5.4 software. Using the GRADEpro GDT software, the quality certainty of each outcome indicator was evaluated according to the GRADE framework. For dichotomous variables, the risk ratio (*RR*) and its 95% confidence interval (*CI*) were used as the effect size when appropriate; for the rare outcome of bile leakage, risk difference (*RD*) was used because the number of events was small and *RD* provides a clinically interpretable absolute effect estimate in sparse-event data. For continuous variables, the mean difference (*MD*) and its 95% *CI* were used as the effect size. Heterogeneity was assessed using the *χ*^2^ test in combination with the I² statistic. If *P* > 0.10 and *I*^2^ < 50%, heterogeneity among studies was considered acceptable, and a fixed-effects model was used; otherwise, a random-effects model was used, and the sources of heterogeneity were analyzed. Sensitivity analyses were conducted using a stepwise exclusion method to assess the robustness of the results for the primary outcome measures. A *P*-value < 0.05 was considered statistically significant.

## 3 Result

### 3.1 Literature screening process and results

The search identified 1,147 records before duplicate removal. After duplicates were removed, 795 records were screened. Following title/abstract screening and full-text assessment, 7 RCTs (4, 6–11) were ultimately included, all of which were published in English. The literature screening process is shown in Figure 1. The quality assessment of the literature is shown in Figures 2, 3.

**Figure 1:**
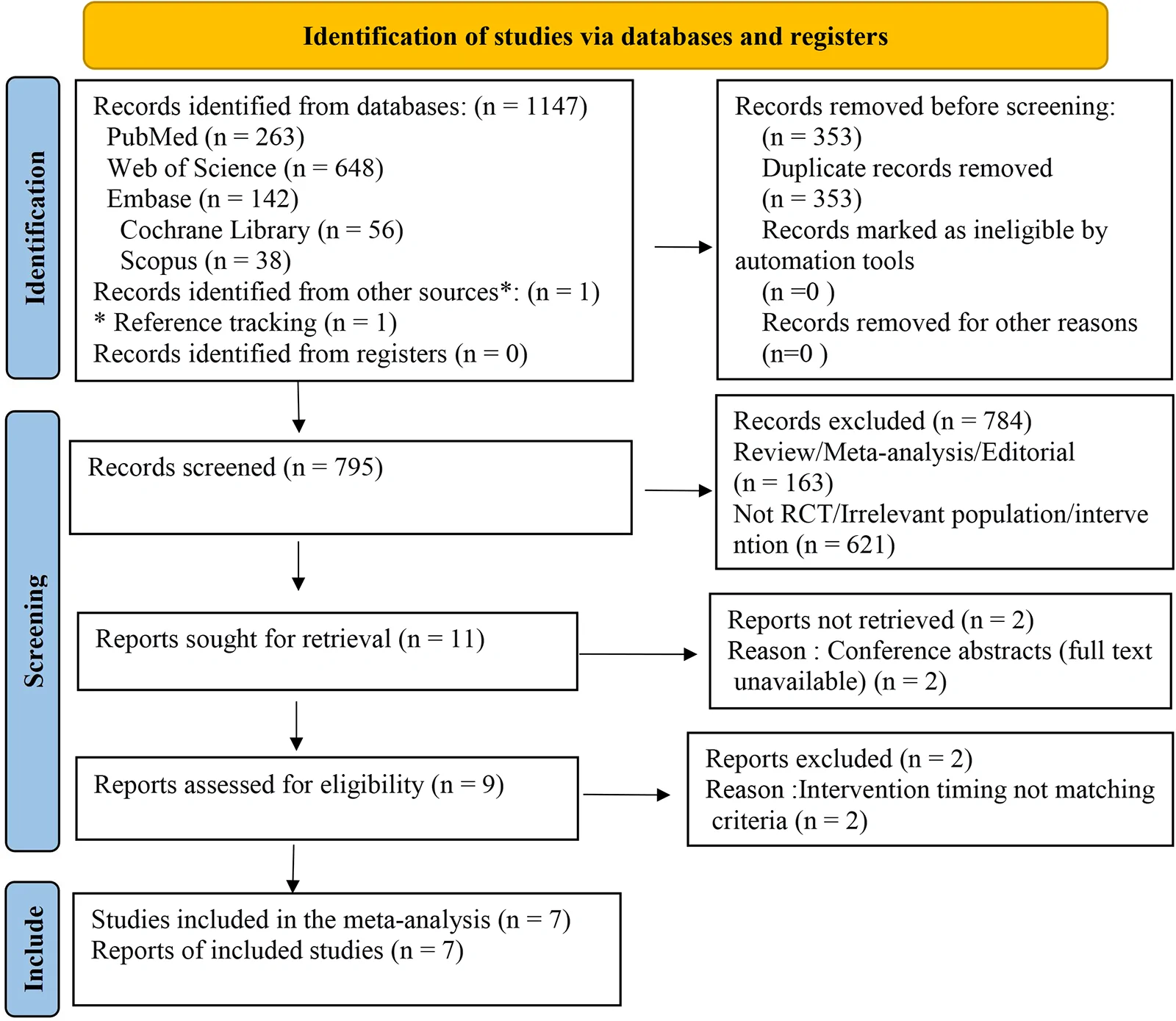
Flow diagram of studies included in the meta-analysis. Of the 7 included studies, 6 were identified through database searching and 1 through reference tracking.

**Figure 2:**
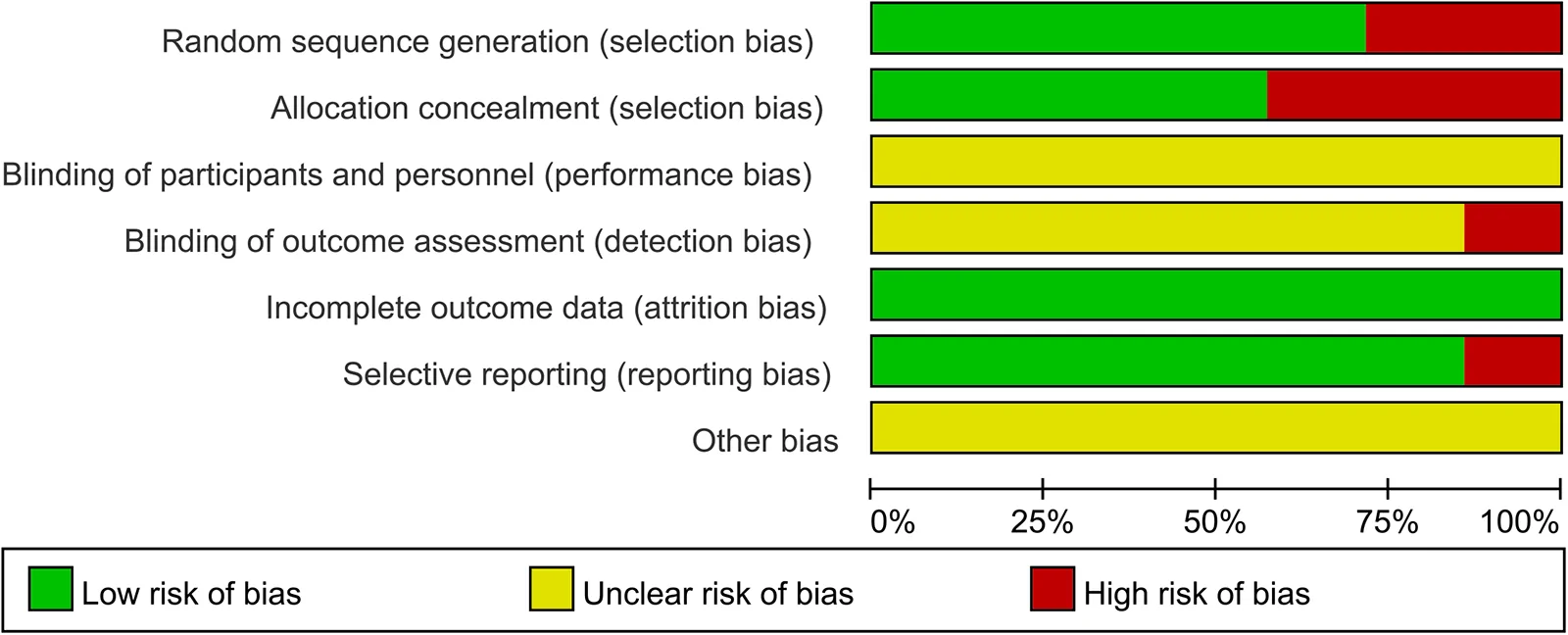
Risk of bias graph.

**Figure 3:**
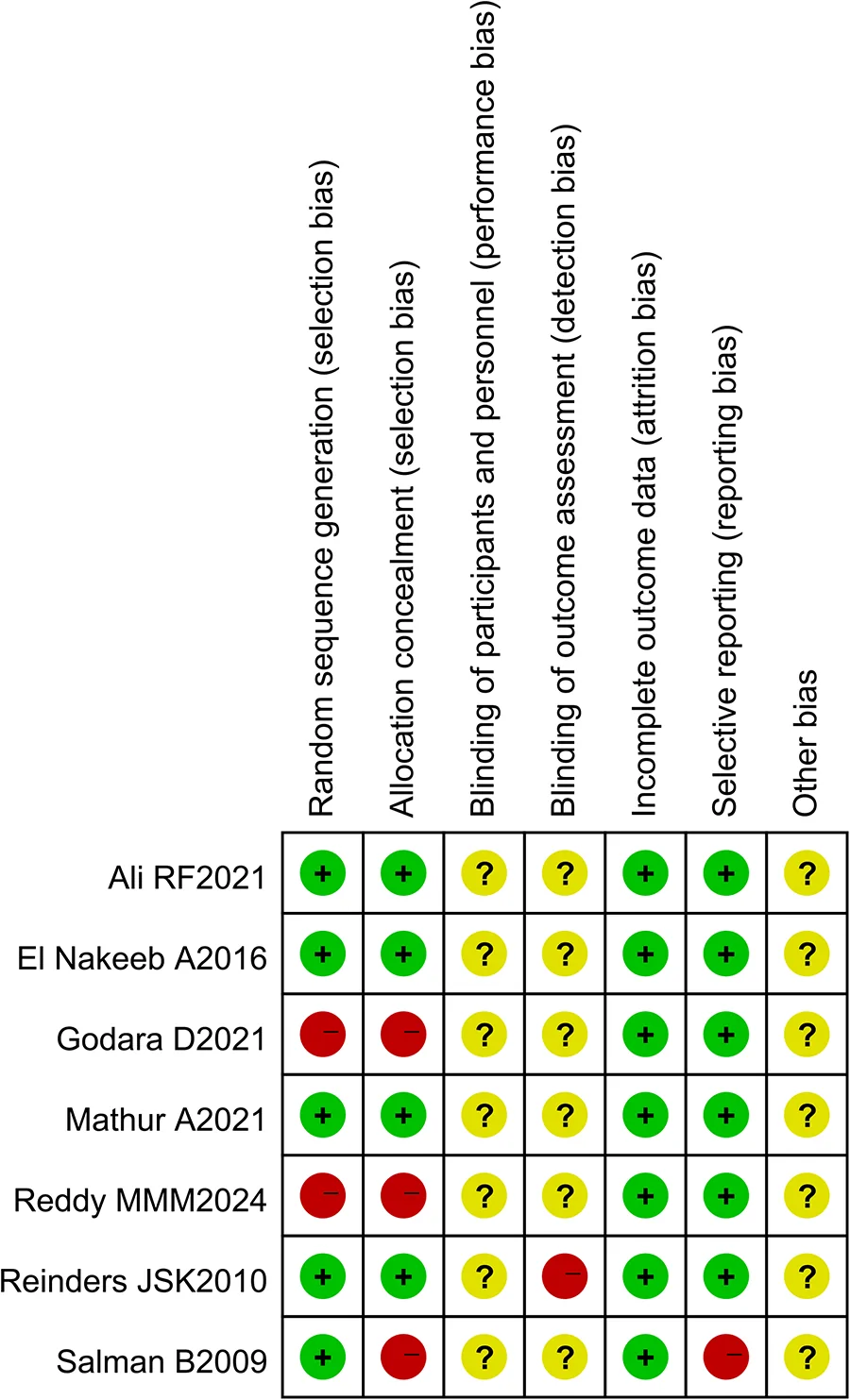
Risk of bias summary.

### 3.2 The basic characteristics included in the study

A total of 7 RCTs were included, involving 593 patients: 296 in the early LC group and 297 in the delayed LC group. Across the included trials, early LC was defined as LC performed within 72 h after ERCP, whereas delayed LC was defined as more than 72 h after ERCP or, in some trials, several weeks later. The reported delayed-LC timing for each trial is summarized in Table 1; where the original study did not provide a more precise interval beyond >72 h, the interval was marked as NR. The risk-of-bias assessment indicated that the main methodological concern was the difficulty of blinding in surgical trials. A GRADE certainty assessment was also conducted for each outcome (Table 2), and the certainty of evidence was interpreted together with heterogeneity and imprecision when drawing conclusions.

**Table 2:** The GRADE summary of findings table includes.

Certainty assessment							No of patients		Effect		Certainty	Importance
No of studies	Study design	Risk of bias	Inconsistency	Indirectness	Imprecision	Other considerations	Early LC	Delayed LC	Relative (95% CI)	Absolute (95% CI)		
New outcome (assessed with: Intraoperative decision to convert to open surgery)												
6	randomised trials	serious^a^	not serious^b^	not serious	not serious^c^	none	10/276 (3.6%)	30/277 (10.8%)	**RR 0.35** (0.17 to 0.68)	**70 fewer per 1,000** (from 90 fewer to 35 fewer)	⊕⊕⊕◯ Moderate^a^^,^^b^^,^^c^	CRITICAL
New outcome (assessed with: Minutes from skin incision to closure)												
5	randomised trials	serious^a^	very serious^d^	not serious	not serious^e^	none	194	195	-	MD **14.37 lower** (28.22 lower to 0.51 lower)	⊕◯◯◯ Very low^a^^,^^d^^,^^e^	IMPORTANT
New outcome (assessed with: Day from LC to discharge)												
5	randomised trials	serious^f^	very serious^d^	not serious	not serious^g^	none	186	184	—	MD **0.81 lower** (1.2 lower to 0.42 lower)	⊕◯◯◯ Very low^d^^,^^f^^,^^g^	IMPORTANT
New outcome (assessed with: Incision infection)New outcome (assessed with: Incision infection)												
5	randomised trials	serious^h^	not serious^i^	not serious	not serious^j^	none	3/216 (1.4%)	18/217 (8.3%)	**RR 0.21** (0.07 to 0.61)	**66 fewer per 1,000** (from 77 fewer to 32 fewer)	⊕⊕⊕◯ Moderate^h^^,^^i^^,^^j^	CRITICAL
New outcome (assessed with: Biliary leakage events)												
3	randomised trials	not serious^k^	serious^l^	not serious	very serious^m^	none	4/177 (2.3%)	13/177 (7.3%)	**RD −0.02** (−0.09 to 0.04)	**– per 1,000** (from–to –)	⊕◯◯◯ Very low^k^^,^^l^^,^^m^	CRITICAL

Bold values indicate the pooled effect estimates (RR, RD, or MD) with 95% confidence intervals from the random-effects or fixed-effects meta-analysis.

CI, confidence interval; MD, mean difference; RR, risk ratio; RD, risk difference.

Explanations.

a It is impossible to blind the surgeon.

b Using the fixed effect model and with heterogeneity *P* > 0.1, the *I*^2^ value is relatively low.

c The 95% confidence interval is 0.17–0.68, which is relatively narrow and does not exceed 1. The sample size of 553 is also relatively large.

d Using the random effects model, the confidence interval width is acceptable, with an *I*^2^ value of 72%, indicating some heterogeneity.

e The 95% confidence interval is −28.22 to −0.51. Although it does not completely cross 0, it is extremely close to 0 (the upper limit is only 0.5 away from 0), and the sample size is only 389, making the estimated value unstable.

f The criteria for discharge vary from hospital to hospital and from country to country.

g The 95% confidence interval is −1.20 to −0.42, which is relatively narrow and does not cross 0.

h It is not clear whether the diagnostician was blinded or not.

i Using the fixed effect model, all research directions are consistent.

j The 95% confidence interval is 0.07–0.61, which is relatively narrow, and the number of events is acceptable.

k Bleeding is an objective judgment (requiring drainage or further intervention), and is relatively less susceptible to bias.

l Using the random effects model and with a wide confidence interval, it is speculated that *I*^2^ is greater than 50%.

m The 95% confidence interval ranges from −0.12 to 0.04, crossing the "no effect line 0", and only three studies were included, with extremely low event numbers and an extremely imprecise estimated value.

### 3.3 The results of the meta-analysis

#### 3.3.1 Conversion rate

A total of 6 RCTs reported conversion rates to open surgery, involving 553 patients. There was no heterogeneity among the studies (*I*^2^ = 3%). A meta-analysis using a fixed-effects model showed that the conversion rate to open surgery was significantly lower in the early laparoscopic cholecystectomy (LC) group than in the delayed LC group (*RR* = 0.35, 95% *CI*: 0.17–0.68, *P* = 0.002) (Figure 4). Sensitivity analyses conducted by sequentially excluding original studies supported the robustness of this result. Because fewer than 10 studies were available, the funnel plot was considered exploratory and was not used to draw a firm conclusion regarding publication bias (Figure 5).

**Figure 4:**
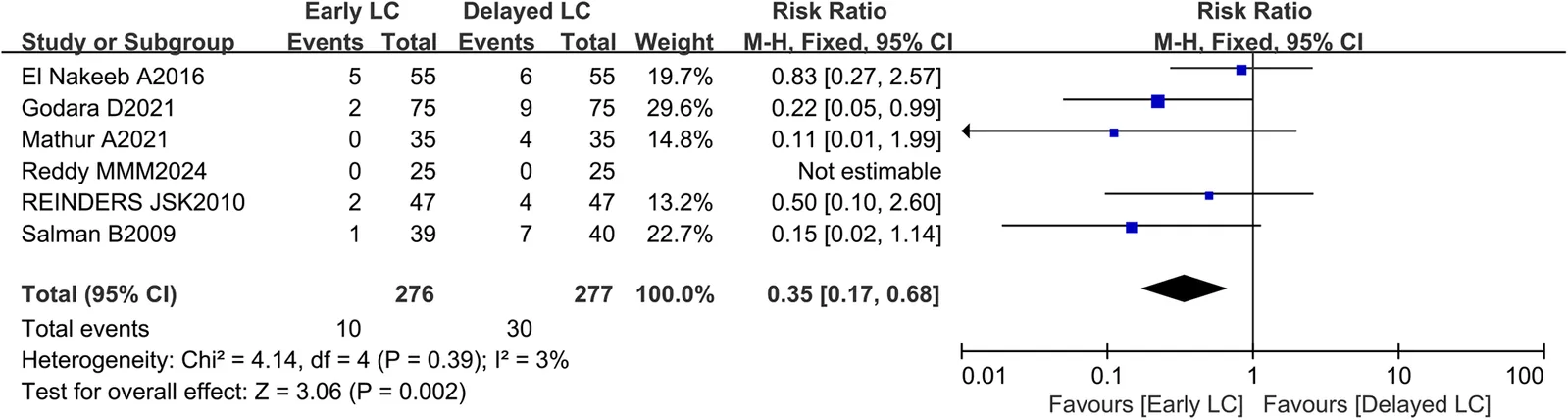
Forest plot of conversion in early and delayed group.

**Figure 5:**
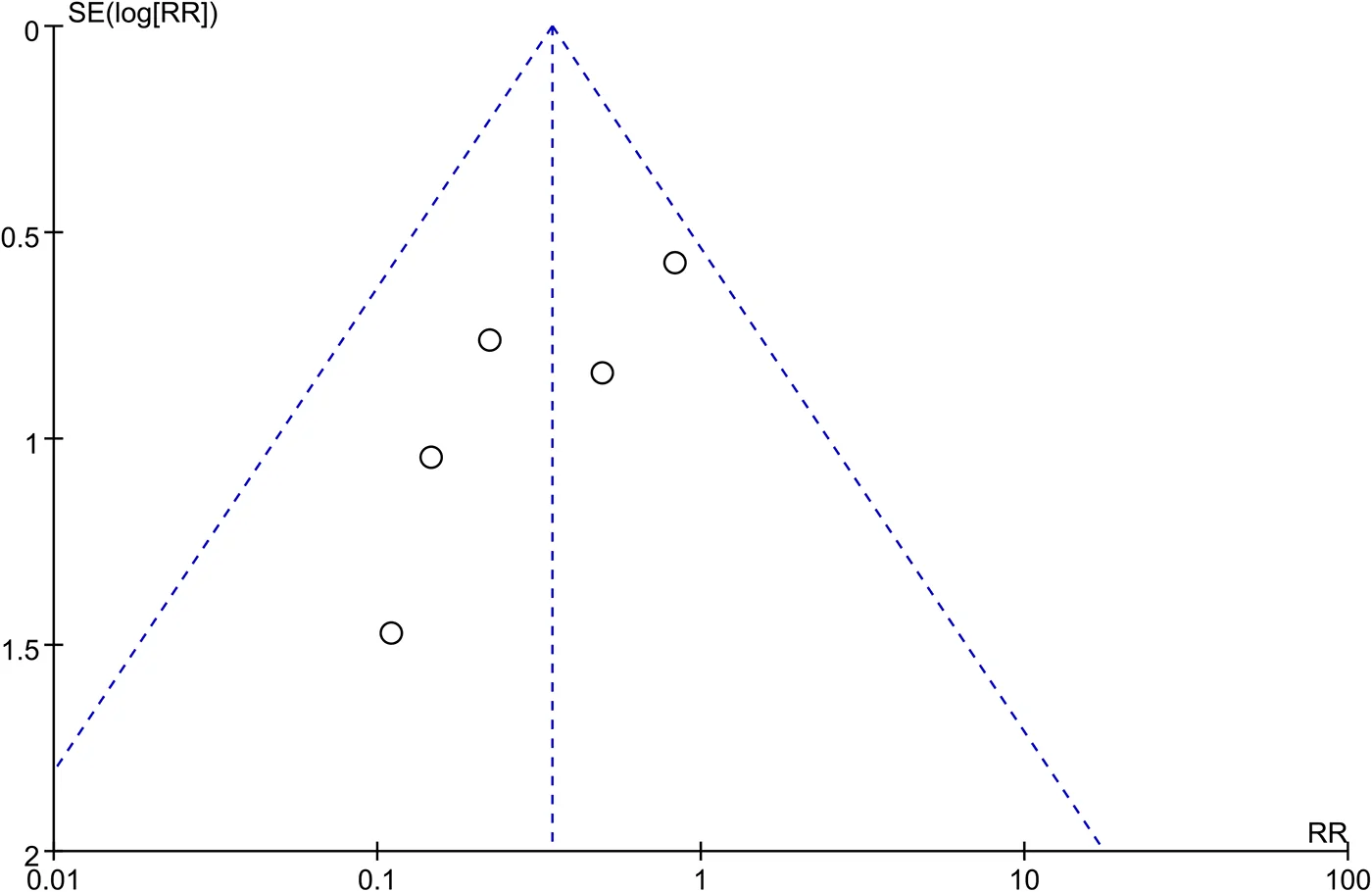
Funnel plot of conversion in early and delayed group.

#### 3.3.2 Operation time

A total of 5 RCTs reported operative time, involving 389 patients. There was high heterogeneity among the studies (*I*^2^ = 96%). A meta-analysis using a random-effects model showed that operative time was significantly shorter in the early LC group than in the delayed LC group (*MD* = −14.37 min, 95% *CI*: −28.22 to −0.51 min, *P* = 0.04) (Figure 6). Leave-one-out sensitivity analysis showed that the direction of effect remained consistent, but the substantial heterogeneity was not driven by any single study, as *I*^2^ remained above 90% after excluding each trial individually. The magnitude of the effect varied across studies; therefore, the reduction in operative time should be interpreted cautiously. Potential sources of heterogeneity include differences in delayed-LC timing, operative-time recording, surgeon experience, and patient anatomical or inflammatory complexity. The funnel plot for operative time is presented as an exploratory analysis only because fewer than 10 studies were included (Figure 7).

**Figure 6:**
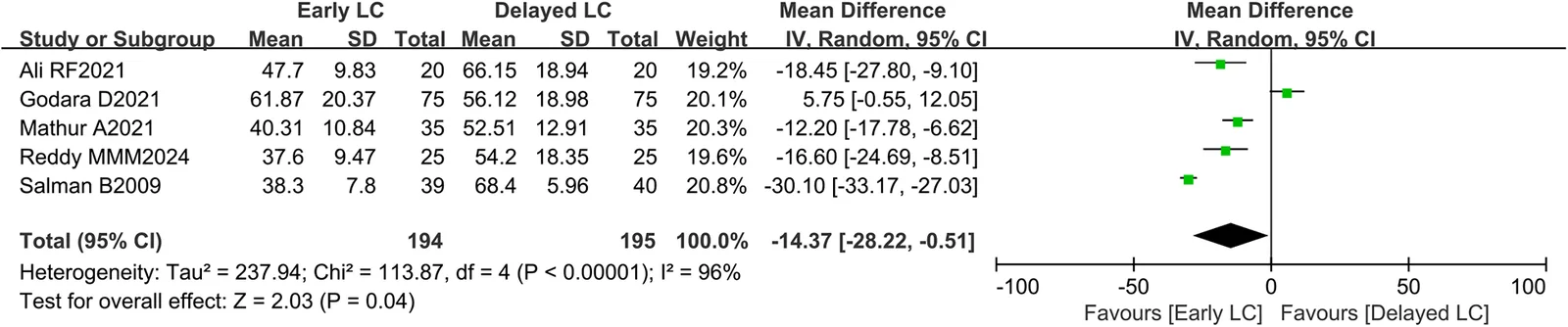
Forest plot of the length of operative time in early and delayed groups.

**Figure 7:**
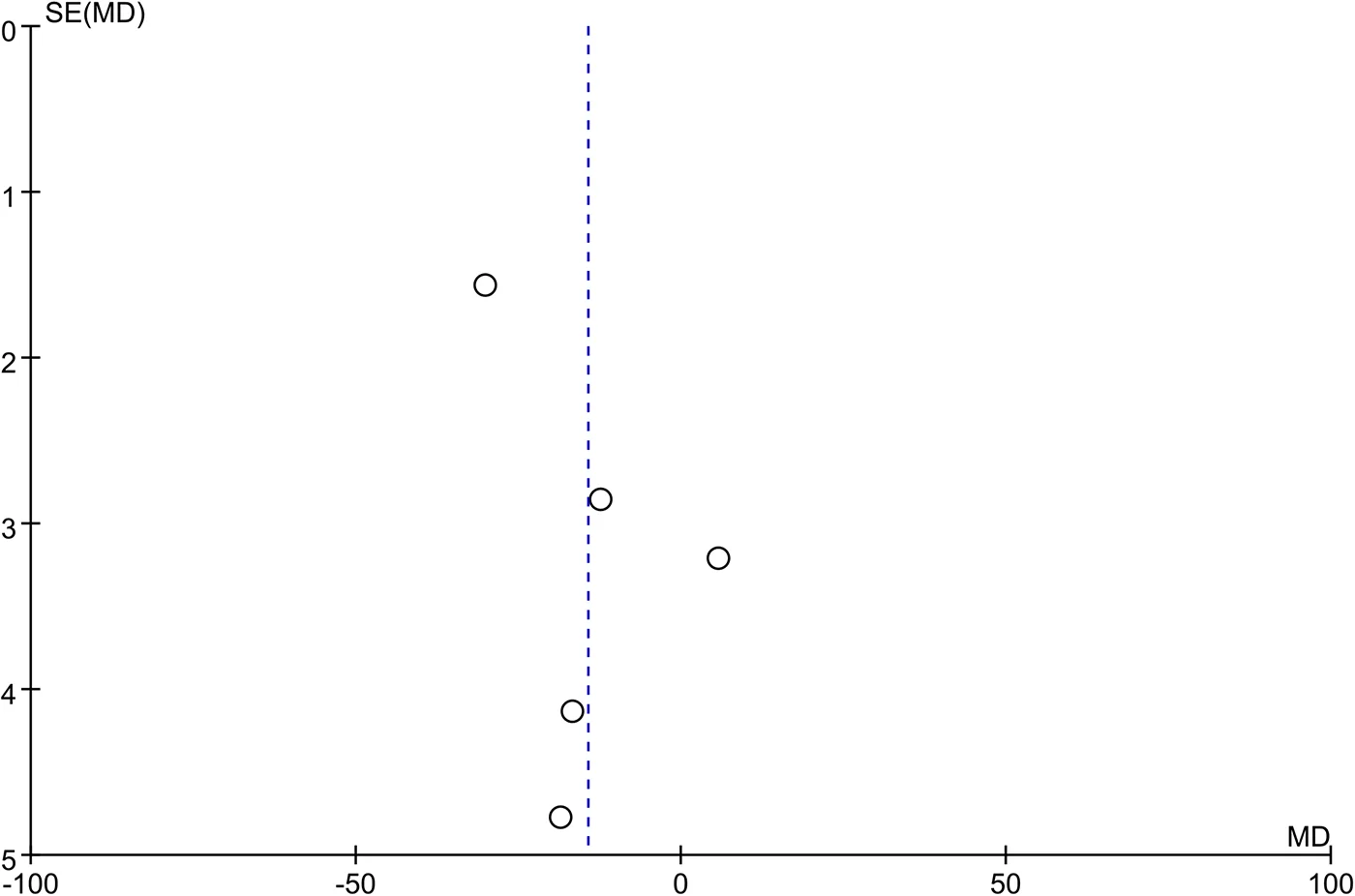
Funnel plot of the length of operative time in the early and delayed groups.

#### 3.3.3 Postoperative hospital stay

A total of 5 RCTs reported postoperative length of hospital stay, involving 370 patients. Moderate-to-high heterogeneity was observed among the studies (*I*^2^ = 72%). A meta-analysis using a random-effects model showed that postoperative hospital stay was shorter in the early LC group than in the delayed LC group (*MD* = −0.81 days, 95% *CI*: −1.20 to −0.42 days, *P* < 0.0001) (Figure 8). Sensitivity analyses showed that the pooled effect remained directionally consistent, with mean differences ranging from −0.66 to −0.95 days across exclusion scenarios. The heterogeneity was partially attributable to the study by Ali RF 2021, whose exclusion reduced *I*^2^ to 53%, suggesting that discharge criteria and healthcare-system differences may contribute to heterogeneity. Because fewer than 10 studies were available, the funnel plot was considered exploratory and was not used to draw a firm conclusion regarding publication bias (Figure 9).

**Figure 8:**
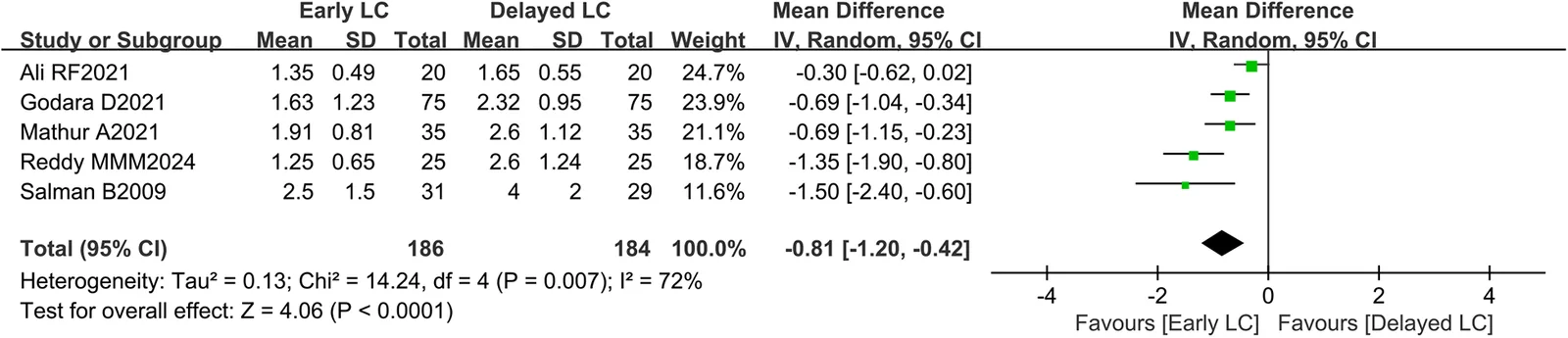
Forest plot of the time of the postoperative hospital stay.

**Figure 9:**
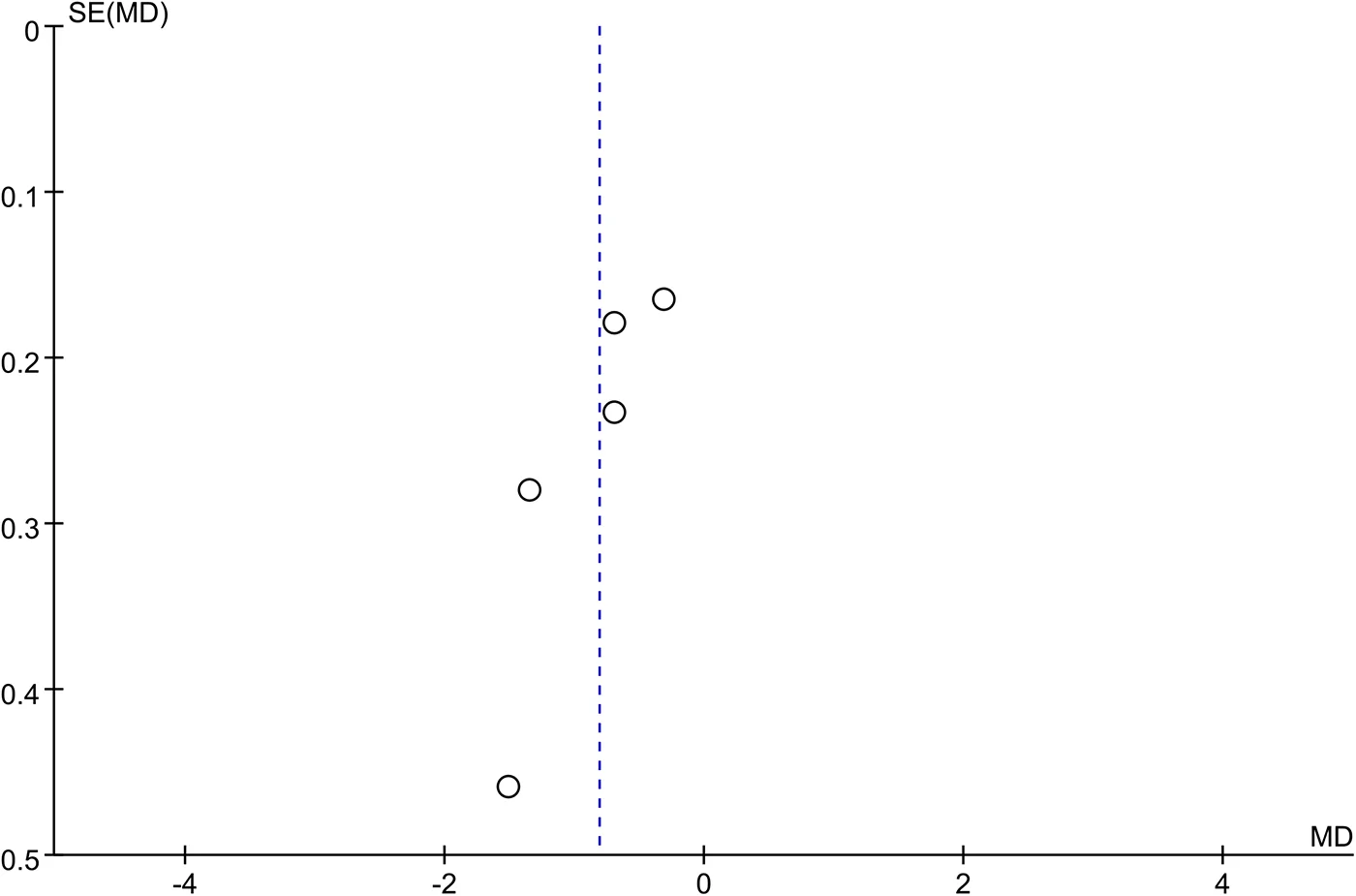
Funnel plot of the time of the postoperative hospital stay.

#### 3.3.4 Incision infection

A total of 5 RCTs reported on incisional infections, involving 433 patients. There was no heterogeneity among the studies (*I*^2^ = 0%). A meta-analysis using a fixed-effects model showed that the incisional infection rate in the early LC group was significantly lower than that in the delayed LC group (*RR* = 0.21, 95% *CI*: 0.07–0.61, *P* = 0.004) (Figure 10). Sensitivity analyses conducted by sequentially excluding original studies supported the robustness of this result. Because fewer than 10 studies were available, the funnel plot was considered exploratory and was not used to draw a firm conclusion regarding publication bias (Figure 11).

**Figure 10:**
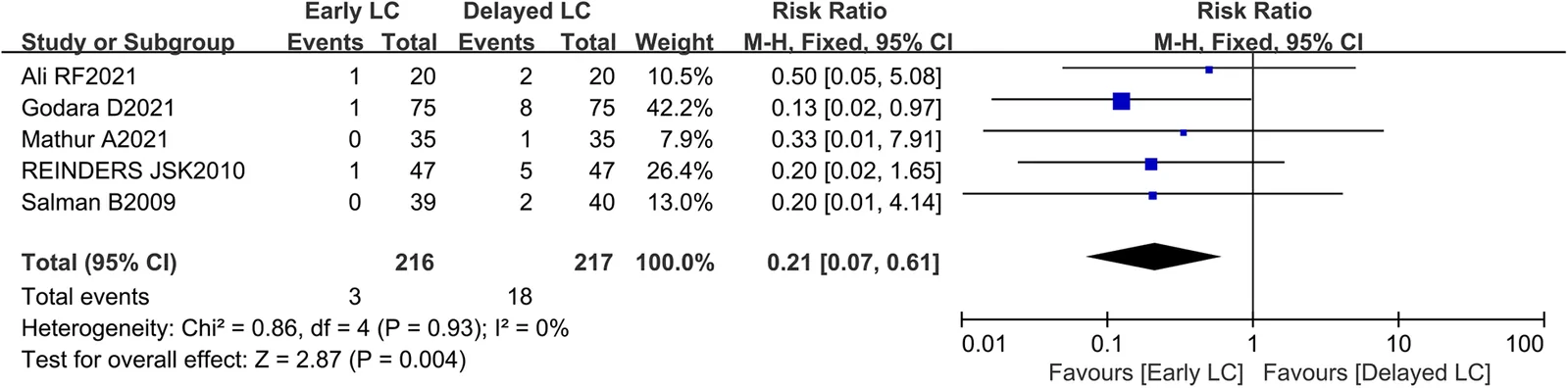
Forest plot of surgical site infection.

**Figure 11:**
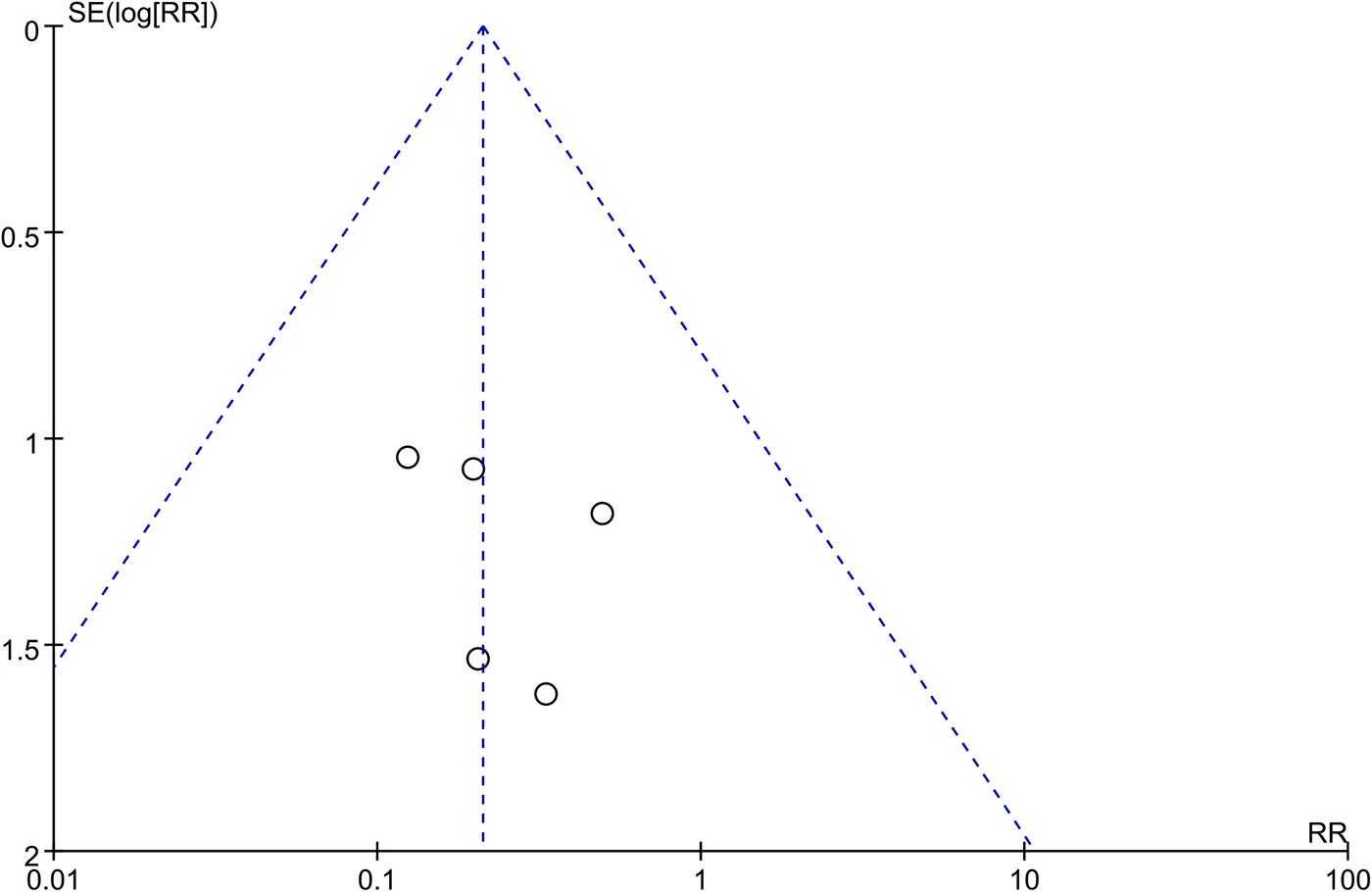
Funnel plot of surgical site infection.

#### 3.3.5 Bile leakage

A total of 3 RCTs reported bile leakage, involving 354 patients. There was moderate heterogeneity among the studies (*I*^2^ = 70%). Using a random-effects risk difference (*RD*) model, the pooled estimate showed no significant difference between the early and delayed LC groups: *RD* = −0.04 (95*% CI*: −0.12 to 0.04; *P* = 0.30) (Figure 12). The absolute event rates were 2.2% (4/177) in the early group and 7.3% (13/177) in the delayed group, indicating a non-significant absolute risk reduction of approximately 4 percentage points associated with early LC. Sensitivity analyses conducted using a stepwise exclusion approach demonstrated that the conclusion of no significant difference between the early and delayed LC groups remained unchanged across all exclusion scenarios. The heterogeneity was partially attributable to the study by Reinders JSK2010, whose exclusion reduced *I*^2^ to 0%. Exclusion of either study did not materially alter the pooled *RD* estimates or the direction of the conclusion. In other studies, it was observed that the *I*^2^ remained above 75% even after excluding each individual study, indicating that heterogeneity stemmed from multiple factors—including differences in definitions of bile leak and variations in routine abdominal drainage practices across trials—rather than from any single outlier study. Only three studies were included, the diagnostic power of the funnel plot is limited, and this conclusion should be interpreted with caution (Figure 13).

**Figure 12:**
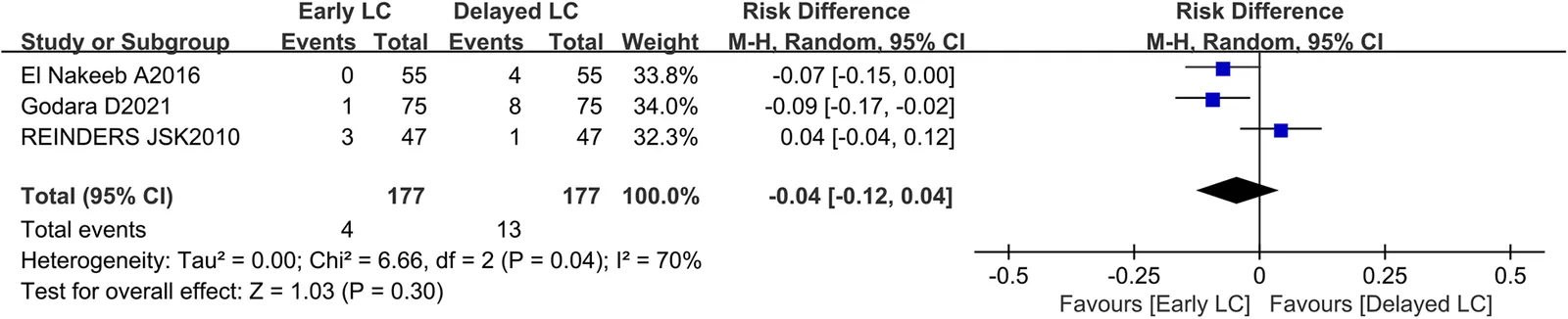
Forest plot of bile leak events.

**Figure 13:**
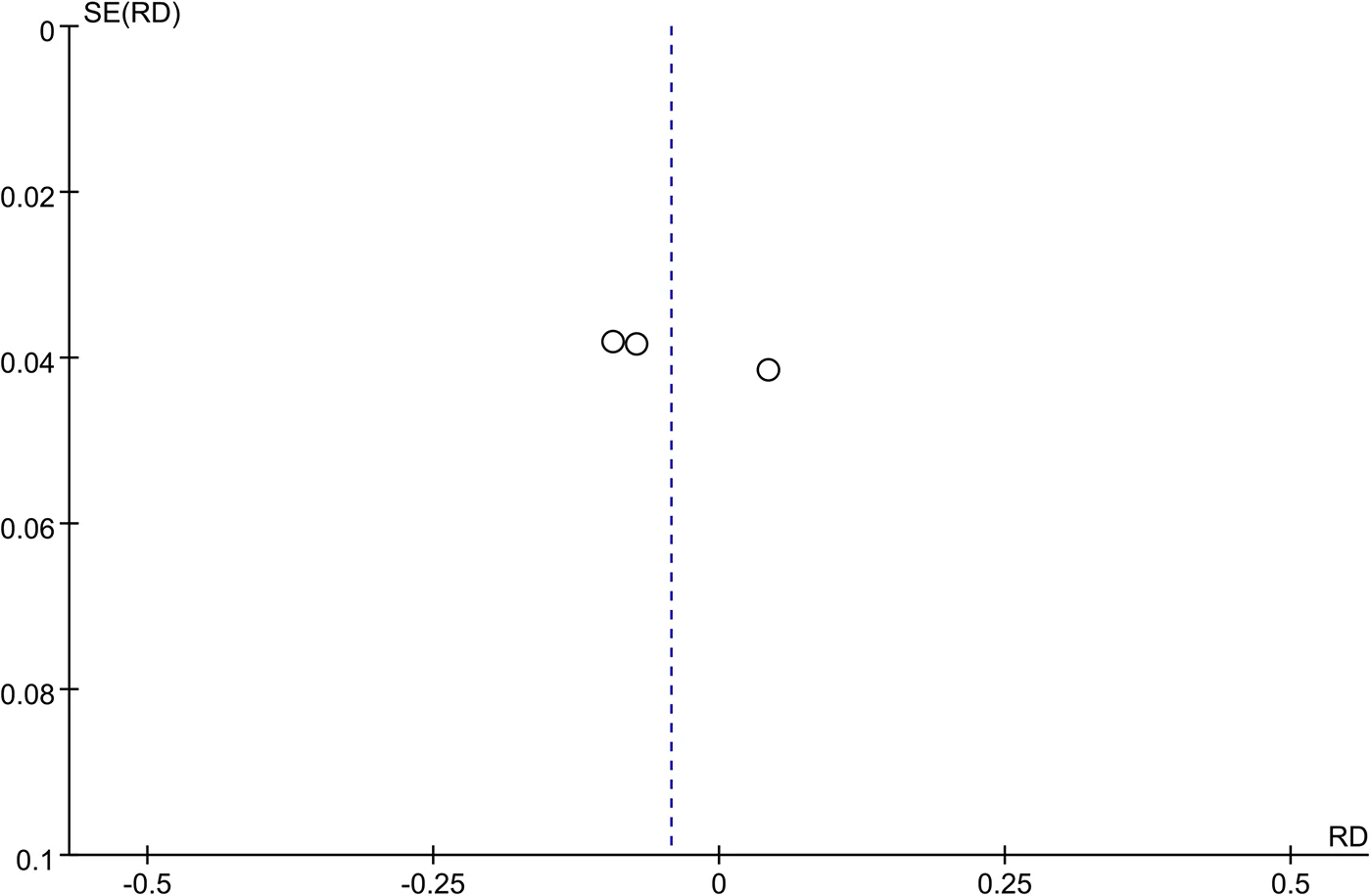
Funnel plot of bile leak events.

## 4 Discussion

This study systematically analyzed the efficacy and safety of early vs. delayed laparoscopic cholecystectomy (LC) following endoscopic retrograde cholangiopancreatography (ERCP) in patients with gallbladder stones and common bile duct stones. A total of 7 randomized controlled trials (RCTs) involving 593 patients were included. The main findings are as follows: (1) Early LC significantly reduced the rate of conversion to open surgery by approximately 65%; (2) Early LC significantly shortened operative time by approximately 14.37 min; (3) Early LC significantly shortened the postoperative hospital stay by approximately 0.81 days; (4) Early LC significantly reduced the risk of surgical site infection by approximately 79%; (5) There was no significant difference between the two groups regarding the serious complication of bile leakage. However, only three studies were included, this conclusion should be interpreted with caution.

### 4.1 Analysis of the advantages of early LC

Previous studies have shown that the conversion rate to laparoscopic cholecystectomy (LC) following endoscopic retrograde cholangiopancreatography (ERCP)/endoscopic sphincterotomy (EST) ranges from 8.7% to 23%, which is significantly higher than that of LC alone (4, 14). This meta-analysis revealed that the conversion rate in the delayed LC group was 10.8% (30/277), whereas it was only 3.6% (10/276) in the early LC group, which was significantly lower than that of the delayed LC group (*RR* = 0.35). The mechanism may lie in the fact that during the period of delayed surgery, recurrent subclinical biliary tract inflammation can lead to fibrosis and increased adhesions in the gallbladder trigone, thereby increasing the difficulty of dissection (15–17); in contrast, early surgery is performed before severe local adhesions have formed, resulting in clearer anatomical planes. A retrospective study by de Vries et al. (18) similarly confirmed that the conversion rate for surgeries performed within 2 weeks after ERCP was significantly lower than for those performed after 2 weeks. Ammar SA et al.'s randomized trial demonstrated that laparoscopic cholecystectomy (LC) performed within 24 h after ERCP is feasible and safe, with a short hospital stay (19). The conclusions of the systematic review and meta-analysis by Wu K et al. (20) indicate that laparoscopic cholecystectomy (LC) performed 24 to 72 h after ERCP offers advantages in terms of conversion rates and is associated with no recurrence of acute cholecystitis, further elucidating the benefits of early LC.

The reduction in both operative time and postoperative length of stay reflects the clinical turnover efficiency of early LC. Although the clinical significance of a 14.37 min reduction in operative time may vary depending on individual differences, when combined with a 0.81-day reduction in length of stay, this holds significant value from a health economics perspective. A randomized controlled trial by Muhammedoglu B et al. (21) confirmed that performing ERCP and LC during the same treatment course and hospital stay effectively reduces costs and shortens the length of stay. The primary benefit is the avoidance of the risk of acute cholecystitis recurrence during delayed cholecystectomy. This approach reduces direct medical costs and alleviates the financial burden on patients to some extent, while also improving bed utilization rates. It is worth noting that the analysis of operative time showed high heterogeneity (*I*^2^ = 96%), which may stem from differences in surgeons' experience across studies, inconsistencies in the definition of “early” (all ≤72 h, but specific time points may vary), and differences in measurement methods. However, the results of the sensitivity analysis indicate consistency in the direction of the random-effects model findings, thereby enhancing the reliability of the conclusions.

The results of this meta-analysis indicate that the incidence of surgical site infection was significantly lower in the early laparoscopic cholecystectomy (LC) group than in the delayed LC group. Possible mechanisms underlying the higher infection rate in the delayed LC group include: patients awaiting surgery frequently sought medical care for biliary colic during the waiting period, increasing the risk of nosocomial cross-infection; recurrent biliary events may lead to occult bacteremia; and prolonged illness may compromise immune function (22–24). A study by Boerma et al. (5) similarly demonstrated that patients in the watch-and-wait group who required emergency surgery due to recurrent biliary events had a significantly higher incidence of infection-related complications.

### 4.2 Safety assessment: there was no significant difference in the occurrence of bile leakage

Bile leakage is one of the most serious complications of concern following laparoscopic cholecystectomy (LC), and its occurrence is closely associated with various factors, including the surgeon's technique in managing the cystic duct and the extent of local inflammation. This meta-analysis showed that the bile leak rate was 2.2% (4/177) in the early LC group and 7.3% (13/177) in the delayed LC group. The pooled estimate did not show a statistically significant difference between groups (*RD* = −0.04, 95% *CI*: −0.12 to 0.04; *P* = 0.30). These data do not suggest an increased risk of bile leakage with early LC; however, only three studies and few events were available, and moderate heterogeneity was present. Therefore, this safety outcome should be interpreted cautiously and requires confirmation in future trials.

### 4.3 Limitations

This study has the following limitations: (1) The limited number of included studies and the small number of events for some outcomes, particularly bile leakage, may reduce statistical power; (2) high heterogeneity was present for operative time, and although sensitivity analyses were conducted, heterogeneity may still reflect differences in surgeon experience, surgical technique, patient complexity, and how operative time was recorded; (3) although early LC was defined as ≤72 h, specific timing within this window may have differed between studies; (4) the delayed group may include clinically heterogeneous scenarios, ranging from more than 72 h to several weeks after ERCP; and (5) postoperative length of hospital stay is influenced by discharge criteria and healthcare systems across countries, so caution is warranted when extrapolating these results.

### 4.5 Implications for clinical practice

Based on the current evidence, early LC during the same hospital stay and within 72 h after successful ERCP may be considered for appropriately selected patients with gallbladder and common bile duct stones. This strategy appears to reduce conversion to open surgery, operative time, postoperative hospital stay, and incisional infection without showing an increased risk of bile leakage. However, because the certainty of evidence varies across outcomes and some analyses show heterogeneity, clinical decisions should also consider patient condition, surgical expertise, and institutional coordination between ERCP and LC. In addition, the applicability of an ERCP-based pathway may depend on local ERCP availability, endoscopic expertise, surgical resources, and institutional coordination. In centers where ERCP is not readily available, non-ERCP strategies for common bile duct stone management should also be considered within the local clinical context (25).

### 4.6 Suggestions for future research

(1) Conduct large-scale, multicenter RCTs to further validate the long-term outcomes of early LC (e.g., recurrence rates, quality of life, and complications); (2) Explore the optimal “early” treatment window (e.g., 24 h vs. 48 h vs. 72 h); (3) Establish uniform diagnostic criteria and severity grading for bile leaks; (4) Conduct health economic evaluations to quantify the cost-effectiveness advantages of early LC; (5) Incorporate patient-reported outcome measures to assess the benefits to early and long-term quality of life from the patient's perspective.

## 5 Conclusion

This meta-analysis of RCTs suggests that early laparoscopic cholecystectomy (LC), defined as within 72 h after ERCP, may offer advantages in reducing conversion to open surgery, shortening operative and postoperative hospital stay, and decreasing incisional infection. For bile leakage, no statistically significant difference was observed between early and delayed LC, but the small number of events and the presence of heterogeneity mean that this safety outcome requires further verification. Overall, early LC appears to be a reasonable same-admission strategy for selected patients with common bile duct stones and gallbladder stones after successful ERCP, while future large-scale RCTs with standardized timing definitions are needed to confirm the magnitude and certainty of benefit.

## Data Availability

The original contributions presented in the study are included in the article/Supplementary Material, further inquiries can be directed to the corresponding author/s.
